# Rheological and Mechanical Behavior of Silk Fibroin Reinforced Waterborne Polyurethane

**DOI:** 10.3390/polym8030094

**Published:** 2016-03-21

**Authors:** Yongzhen Tao, Anwarul Hasan, George Deeb, Changkai Hu, Huipeng Han

**Affiliations:** 1 Key Laboratory of Green Processing and Functional Textiles of New Textile Materials, Ministry of Education, Wuhan Textile University, Wuhan 430073, China; changkaihu1119@163.com (C.H.); hhp950324@163.com (H.H.); 2 Department of Mechanical and Industrial Engineering, College of Engineering, Qatar University, Doha 2713, Qatar; 3 Biomedical Engineering, and Department of Mechanical Engineering, Faculty of Engineering and Architecture, American University of Beirut, Beirut 1107 2020, Lebanon; gdd01@mail.aub.edu; 4 Biomaterials Innovation Research Center, Division of Biomedical Engineering, Department of Medicine, Brigham and Women’s Hospital, Harvard Medical School, Cambridge, MA 02139, USA; 5 Harvard-MIT Division of Health Sciences and Technology, Massachusetts Institute of Technology, Cambridge, MA 02139, USA

**Keywords:** waterborne polyurethane, silk fibroin powder, rheological behavior, reinforcement

## Abstract

Waterborne polyurethane (WPU) is a versatile and environment-friendly material with growing applications in both industry and academia. Silk fibroin (SF) is an attractive material known for its structural, biological and hemocompatible properties. The SF reinforced waterborne polyurethane (WPU) is a promising scaffold material for tissue engineering applications. In this work, we report synthesis and characterization of a novel nanocomposite using SF reinforced WPU. The rheological behaviors of WPU and WPU-SF dispersions with different solid contents were investigated with steady shear and dynamic oscillatory tests to evaluate the formation of the cross-linked gel structure. The average particle size and the zeta potential of WPU-SF dispersions with different SF content were examined at 25 °C to investigate the interaction between SF and WPU. FTIR, SEM, TEM and tensile testing were performed to study the effects of SF content on the structural morphology and mechanical properties of the resultant composite films. Experimental results revealed formation of gel network in the WPU dispersions at solid contents more than 17 wt %. The conjugate reaction between the WPU and SF as well as the hydrogen bond between them helped in dispersing the SF powder into the WPU matrix as small aggregates. Addition of SF to the WPU also improved the Young’s modulus from 0.30 to 3.91 MPa, tensile strength from 0.56 to 8.94 MPa, and elongation at break from 1067% to 2480%, as SF was increased up to 5 wt %. Thus, significant strengthening and toughening can be achieved by introducing SF powder into the WPU formulations.

## 1. Introduction

Waterborne polyurethane (WPU) is a widely used versatile class of material for applications in coatings, adhesives, sealants, elastomers, porous-foams, biomaterials and many more [1,2,3,4]. Polyurethane has been used as scaffold material in tissue engineering, and as a career material in drug delivery as well [5]. While there are two types of polyurethane dispersions, namely solvent-borne and waterborne, the waterborne ones are particularly attractive due to their higher durability, better environment-friendliness and better tailoring capabilities. The rheological behavior [6,7,8] of WPU dispersions and the mechanical properties [9,10,11] of WPU films are of paramount importance for controlling their flow during applications in coating of textile fibers, sealants, and glass fiber sizing, as well as controlling the mechanical properties of solids prepared from these dispersions [12]. Polymer composites [13,14], particularly those made of WPU, have been subjects for rigorous investigations in the domains of industry and academia due to their simplicity and effectiveness in obtaining new materials [15,16]. More specifically, polymer composites reinforced with microscale or nanoscale fillers have received huge attention owing to their unique and fascinating properties that potentially rival those of most advanced natural materials [17,18]. In recent years, various natural polymers have been used in the development of different types of hydrogels that have been playing crucial roles in the field of tissue engineering, from improved cell growth and proliferation in three dimensional cell culture to improved application of nano-scale biosensors and the enhancement of their performance [19,20].

Silk fibroin (SF) is a natural fiber, made of a group of proteins that are multiblock copolymers containing crystallizable and uncrystallizable blocks [21]. SF is attractive for its structural properties as well as its biological and hemological compatibility [22]. The semi-crystalline SF can act as pattern to guide the formation of more ordered pack of hard segments of WPU. Furthermore, SF is amply found in hydrophobic amino acid residues. It can also be found to a lesser degree in some hydrophilic amino acids by virtue of their hydroxyl, carboxyl and amino groups, which are ready to react with the residual isocyanate groups (–NCO) of WPU prepolymer [23,24].

Recently, synthesis of WPU-SF dispersions and the high-strength composite materials of WPU-SF have been reported [25,26]. WPU dispersions belong to a distinct group of colloidal dispersions that are known to exhibit complex rheological behavior [27,28]. The rheological behavior of WPU and WPU-SF dispersions is not yet well understood. However, it is important to clarify the rheology properties of the dispersions, which can provide a strong and essential basis and framework for the optimization of the application and function of WPU-SF in response-driven polymer coating and film formation [14].

In this work, the dispersions of WPU and WPU-SF with various solid contents were synthesized for preparing the novel composite WPU-SF materials with enhanced physical and biological properties. The solid contents of WPU were set in the range from 13 to 25 wt %, and the SF powder with 3 μm diameter was incorporated during the synthesis of the 17 wt % WPU to prepare WPU-SF dispersions with different SF content (1–5 wt %). The rheological behavior, the size of the particles, and the zeta potential of the WPU-SF dispersions were investigated. Furthermore, the resultant WPU-SF dispersions were then used to prepare composite films, and the effect of SF content on the structure, morphology, and mechanical properties were discussed.

## 2. Experimental

### 2.1. Materials

The Poly(oxypropylene glycol) (PPG, *M*_n_ = 1980 g/mol) and 2,4-Toluene diisocyanate (TDI) were obtained from Jiangbei Chemical Reagents Factory (Wuhan, China). Acetone and Triethylamine (TEA) were obtained from Shanghai Chemical Co. (Shanghai, China). Dimethylol propionic acid (DMPA) was received from Chengdu Polyurethane Co. (Chengdu, China). Raw silk filament was received from Luotian Silk Incorporation (Huanggang, China). The DMPA and PPG were vacuum-dried and TDI was redistilled, before using. Acetone and TEA were dehydrated using 3 Ǻ molecular sieves.

### 2.2. Preparation of SF Powder

Raw silk filament obtained from Luotian Silk Incorporation was degummed, dried and pulverized to obtain the powder with a diameter of around 3 μm. The preparation of the SF powder was described in detail in the earlier work [25,26]. In brief, the sericin was removed from the silk fibroin by boiling the raw silk filament at 95 °C for 1 h twice in a 0.3 M sodium carbonate solution. Thus, the silk filament was degummed, which enabled thoroughly washing the filaments in warm distilled water, and then allowing them to dry at 50 °C in a ventilated oven. As the final step, the silk fibroin powder of around 3 μm diameter was obtained by pulverizing the washed and dried silk filament in a custom-made pulverizing machine [29].

### 2.3. Synthesis of WPU

WPU aqueous dispersions with different solid contents were prepared as described in our previous work [25,26]. At first a four-neck glass flask of volume 500-mL was equipped with a heating mantel and a nitrogen supply line. About 1.58 g of DMPA and about 23.60 g of PPG were added to the flask and heated to a temperature of 80 °C. The mixture was continuously agitated for 1 h using a magnetic stirrer. About 7.2 g of TDI was added slowly drop-by-drop, and the reaction was left to proceed for 3 h under dry nitrogen atmosphere. The temperature was cooled down to 40 °C, the viscosity was reduced by adding acetone in the reactor. The—COOH groups of DMPA were neutralized by adding TEA, and the mixture was vigorously stirred for 1 h in order to disperse it in distilled water. To finish, the stirring at ambient/room temperature was continued overnight and various groups of WPU dispersions with different solid contents were prepared by adding desired amount of distilled water, and coded as WPU13, WPU15, WPU17, WPU20, WPU22, and WPU25, corresponding to solid contents of 13, 15, 17, 20, 22, and 25 wt %, respectively.

### 2.4. Preparation of the WPU17-SF Dispersions and Films

To obtain the WPU17-SF dispersions, PPG, DMPA, and various proportion of SF powder were added into the reactor for the 17 wt % WPU prepolymer dispersions according to the synthesis steps mentioned above. The formulations are shown in Table 1. The WPU17-SF dispersions with different amounts of SF were weighed and kept in vacuum for 15 min to get rid of any air that was still present. Air removal was important for avoiding formation of bubbles during the drying process. The dispersions were then placed in glass square Petri dishes and were left to dry for 7 days at ambient temperature. WPU17-SF films of about 0.7 mm thickness were prepared and named as WPU17, WPU17-SF1, WPU17-SF2, WPU17-SF3, WPU17-SF4, and WPU17-SF5, in accordance with the SF content of each film (0, 1, 2, 3, 4, and 5 wt %, respectively). Furthermore, the films were exposed to a temperature of 50 °C for 12 h in a ventilated oven to dry. The films were then placed in a desiccator, which contained P_2_O_5_ with 0% relative humidity at room temperature for preconditioning prior to various characterizations.

### 2.5. Measurement of Rheological Behavior

Steady shear and dynamic oscillatory tests [7,8,30] were performed for characterizing the rheological properties of the WPU dispersions with different solid contents as well as the WPU17-SF dispersions with different SF contents. An AR 2000ex rheometer (TA Instrument, New Castle, DE, USA) was used to perform all the tests. The double-concentric cylinder geometry with a 4 mm gap was used to measure the parameters under the steady shear flow procedure at 25 ± 0.2 °C. The dynamic oscillatory tests were conducted by using a 40 mm steel parallel geometry and 1 mm gap. Temperature sweep for the WPU17-SF5 dispersion was performed at 1 Hz and 1% strain upon heating from 20 to 70 °C at a rate of 1 °C /min. Time sweep was used to monitor the crosslinking process of the WPU17-SF5 dispersion at 37 °C at 1 Hz and 1% strain for 2 h. Dynamic frequency sweep was performed in the frequency range from 0.1 to 200 rad/s at 37 °C by keeping the strain constant at 1% (within the linear viscoelastic region). The storage modulus (*G′*), the loss modulus (*G*′′), tan δ, and the complex viscosity (η*) were recorded as a function of temperature, time, and angular frequency (ω), respectively. The existence and extent of the linear viscoelastic regime was determined by plotting *G′* and *G*′′ as a function of strain (which varied from 0.1% to 100%) at a constant angular frequency of 6.28 rad/s.

### 2.6. Measurement of Particle Size and Zeta Potential

A particle sizer (Malven Instruments, Worcestershire, UK) and a zeta potential analyzer (Nano-ZSZEN3600, Malven Instruments, Worcestershire, UK) were used to measure the diameter of the particles and the zeta potential for the WPU17, and WPU17-SF dispersions with varying SF contents at 25 °C.

### 2.7. FTIR Spectroscopy

A Nicolet ATR-FTIR spectrometer (AVATAR 360, Pittsfield, MA, USA) with a germanium (Ge) crystal was used to perform ATR-FTIR spectroscopy at room temperature and a 45° incident angle. After placing the samples flatly on the crystal surface, a resolution of 4 cm^−1^ was used to conduct 16 scans for data collection. To record the background spectra, sweeps were conducted with the samples absent keeping the Ge crystal only in contact with air.

### 2.8. SEM and TEM Imaging

A scanning electron microscope (SEM, S-570, Hitachi, Japan) at a voltage of 20 kV was used to observe the morphology of the films after sputtering the fractured surface with gold. A JEM-2020 TEM (JEOL TEM, Tokyo, Japan) was used to observe the morphology of the WPU17 and the WPU17-SF3 films. In brief, EPon 812 epoxy resin was used to embed the WPU17 and the WPU17-SF3 film which were then cured at 40 °C for 60 h. A razor blade was first used to trim the embedded specimens. A glass knife equipped ultra-cut microtome was then used to further trim the specimens. Using the cross-section of the polymer strip enabled the achievement of a trapezoidal top surface which was extremely smooth. For ultra-thin microtomy, an ALKBIII microtome was used. A glass knife was used to remove the top layer (about 1 mm). A Diatome diamond knife was then used to cut ultra-thin sections of about 100 nm were cut with at room temperature. OsO_4_ was used to stain the ultra-thin sections which were then mounted on 200 mesh copper grids. In the end, a TEM operating at an accelerating voltage 200 kV was used to examine the sections.

### 2.9. Measurement of Mechanical Properties

A universal testing machine (Instron 5566, Shakopee, MN, USA) with 1.5 cm gauge length and 100 mm/min displacement rate was used to measure the mechanical properties of the films. The tensile strength (σ_b_), extent of elongation at failure (ε_b_), as well as the Young’s modulus (*E*) of the films were computed from the tensile stress-strain graphs. The average obtained from a minimum of five test runs per sample was used for computing the mechanical properties of the samples.

### 2.10. Statistical Analyses

Data were analyzed using analysis of variance (ANOVA). Results were considered statistically significant when *p* < 0.05. The calculations were performed using Origin Software (Version 6.1, Northampton, MA, USA).

## 3. Results and Discussion

**Figure 1:**
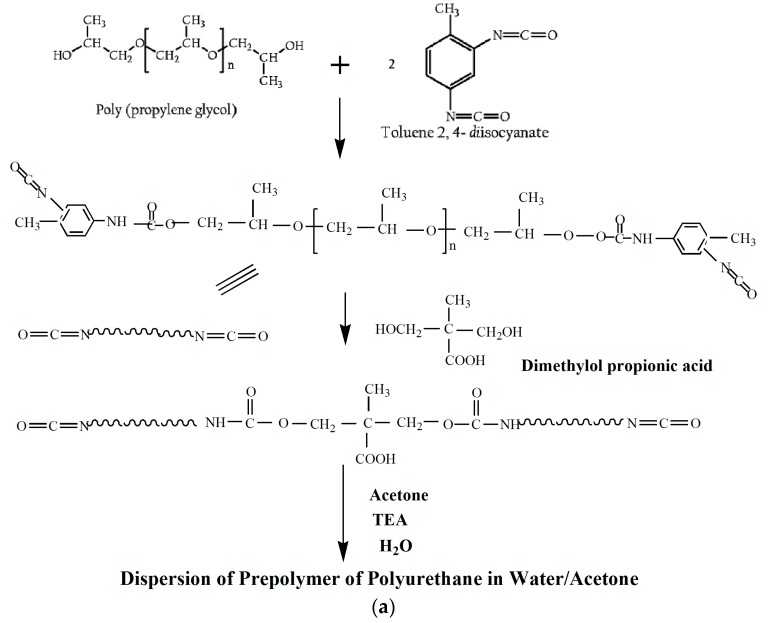

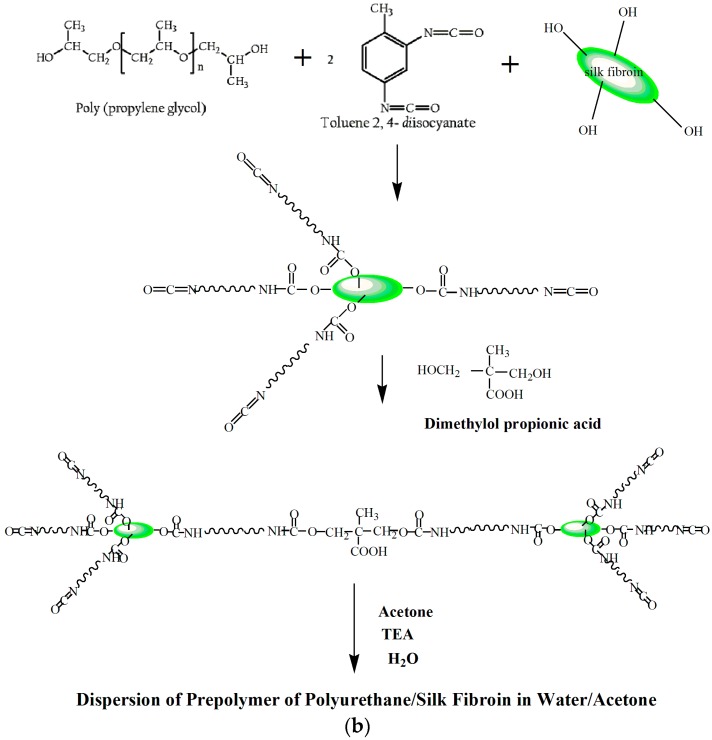
Schematic representation of the preparation of: (**a**) WPU Prepolymer and (**b**) the WPU-SF dispersions in Water/Acetone.

In this work, a novel composite using silk fibroin in WPU dispersion is prepared and studied. The rheological behavior of WPU and WPU-SF dispersions was investigated to understand the flow behavior and network architectures of WPU and WPU-SF dispersions and films at different solid contents. The synthesis of the WPU dispersion is shown in Figure 1a. Since the polyurethane in the WPU dispersions are multi-block copolymers with different chemical species, the interfacial forces and physiochemical interactions play an important role in determining the stability and behavior of the dispersions. In order to understand the variation in the rheological behavior and the mechanical properties of WPU-SF dispersions and films due to the addition of powdered SF, five concentrations of SF were incorporated into the WPU formulation. The hydroxyl groups on the surface of SF powder were hypothesized to react with the isocyanated groups of WPU prepolymer, as shown in Figure 1b. Due to the thermodynamic incompatibilities arising from the repetition of alternating soft and hard segments in WPU-SF, biphasic morphology is formed. An increase in the hard microdomains leads to an increase in the stiffness while an increase in hydrogen bond interactions between the soft and hard segments increases the elasticity as will be discussed in subsequent sections.

### 3.1. Steady-Shear Flow Behavior of WPU Dispersions

**Figure 2:**
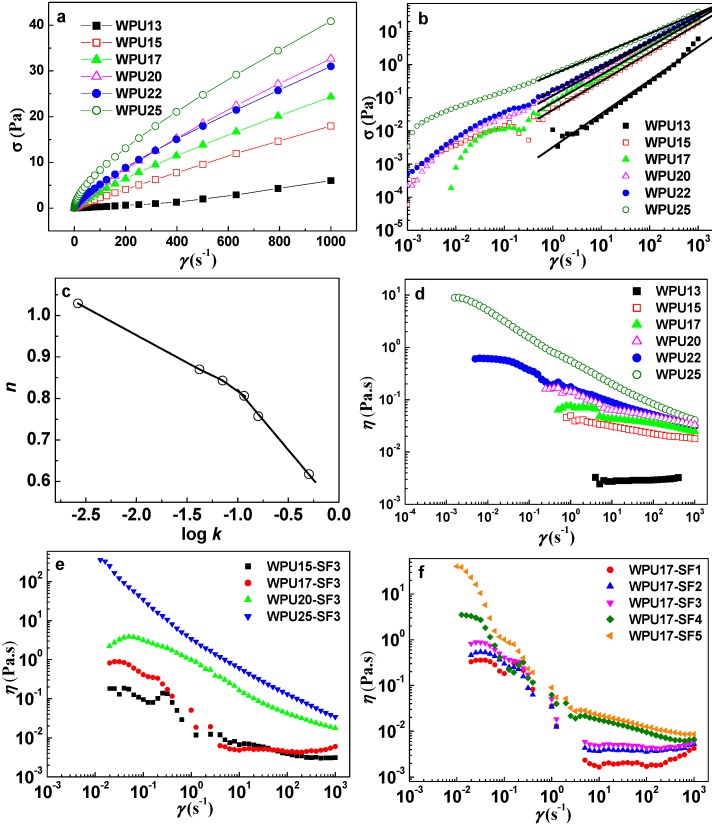
Steady-shear flow behavior of WPU dispersions: dependence of the shear stress σ on the shear rate γ (**a**,**b**); flow behavior index, *n*, as a function of log *k* (**c**); and dependence of the steady shear viscosity on the shear rate for WPU dispersions with different solid contents (**d**); for WPU-SF3 dispersions (**e**); and for WPU17-SF (**f**) at *T* = 25 °C, respectively.

Relationships between shear stress and shear rate in fluids can be categorized as Newtonian, pseudo-plastic, Bingham plastic, Bingham, and dilatants behavior. For the Newtonian, the shear stress is directly proportional to shear rate, which results in constant viscosity. The other types of fluid behavior are called non-Newtonian fluids, and the viscosity could be varied with shear rate. The steady shear flow states of the WPU dispersions with different solid contents are shown in Figure 2a. At very low solid content (13 wt %), the shear stress (σ) is linear with respect to shear rate (γ), indicating Newtonian behavior of the polyurethane water dispersions with this solid content. However, for dispersions with a higher solid content, the correlation between shear stress and shear rate is non-linear and can be explained by using the pseudo-plastic behavior. A yield point, which is a transition point to a plastic flow, shows the binding strength of the network structure in the WPU dispersions. The shear stress at the yield point was obtained by extrapolating shear stress *vs* shear rate curves as illustrated in Figure 2b. The yield stress of WPU15, WPU17, WPU20, WPU22, and WPU25 was 0.021, 0.032, 0.084, 0.110, and 0.386 Pa respectively. The yield stress increases with the increase of WPU solid content, and has a relatively significant increase beginning at WPU20. High yield stress is mainly due to the formation of gel structure in WPU with solid content more than 17 wt %. The effect of the WPU solid content on the shear rate–shear stress relationship was further studied, and the data were fitted with Ostwald-de Waele Equation given by [31].

(1) $$\sigma =k\gamma^{n}$$

where σ is the shear stress (Pa), γ is the shear rate (s^−1^), *k* (Pa s*^n^*) is the flow consistency index and *n* is the flow behavior index. The flow behavior index “*n*” and the flow consistency index “*k*” are constant at a given temperature since they are among the properties of the fluid. A log-log plot of σ *versus* γ results in a slope *n* (the power-law exponent), where *n* = 1 describes Newtonian fluid behavior, *n* < 1 indicates pseudo-plastic behavior, and *n* > 1 corresponds to shear-thickening behavior. By fitting the steady shear flow data (Figure 2b), an *n* value of 1.03 was obtained for the 13 wt % WPU dispersion indicating that the dispersion is a dilute Newtonian fluid. However, for dispersions with a higher solid content, the *n* values were less than 1, which gradually decreased from 0.87 for 15 wt % WPU to 0.62 for 25 wt % WPU. Thus, the polyurethane dispersions in 15–25 wt % solid content range exhibited a pseudo-plastic behavior.

Using the Ostwald-de Waele equation, values of *n* as a function of log *k* were obtained and are shown in Figure 2c. The *n* value was a linearly decreasing function of log *k*, further confirming a divergence from the behavior of typical Newtonian liquids to a pseudo-plastic behavior. The *n* –log *k* plot exhibited two distinct parts. The first part showed a slow decrease of *n* with log *k*, and it lies in the solid content range of 13 to 17 wt %, with a slope of −0.13. Whereas, in the second part, where solid content is higher than 17 wt %, the *n* value decreases considerably with the increase of log *k*, showing a slope of −0.29. The results are in agreement with literature, asserting that the slope of the *n*–log *k* plot varies with concentration, temperature, and blending of polymer solution [32]. The results further revealed that the shear thinning behavior became more pronounced as the solid content of WPU dispersion increased.

The dependence of steady shear viscosity (η) of WPU dispersions on the shear rate at 25 °C is shown in Figure 2d. Clearly, when the solid content was low (13 wt %), the WPU dispersion exhibited Newtonian fluid behavior, and the viscosity, η, remained constant. As the solid content increased, the dispersion exhibited shear-thinning behavior. This suggests an enhancement of the chain entanglement of WPU in the quiescent dispersions with high solid contents, and a disruption of the chain entanglement of WPU with an increase in shear rate. At low shear rates, the existence of a Newtonian plateau for η was evident, and the range of plateau shifted to lower shear rates as the solid content of WPU dispersions increased. Thus the results suggest that the critical shear rate (γ_c_) corresponding to the transition from Newtonian to behavior shifts to a lower shear rate with an increase of the solid content of WPU. The increment in viscosity is also accompanied by a shift to a lower critical shear rate. This suggests that the relative high viscosity and low critical shear rate may be dependent on the formation of network structure in WPU dispersions as solid content is higher than 17 wt %.

The dependence of shear rate on the viscosity of the WPU-SF3 dispersions is shown in Figure 2e. The WPU25-SF3 and WPU20-SF3 samples exhibit shear thinning almost over the whole shear rate range investigated, whereas the viscosity-shear rate profile changes to one exhibiting multi-distinct regions with a shear-thickening behavior at intermediate shear rate for the WPU17-SF3 and WPU15-SF3 samples. This multi-region viscosity profile consists of a plateau region followed a shear thinning at low shear rates due to the alignment of the WPU-SF composite particles; a shear-thickening region at intermediate shear rates, where the SF particles act as cross-linkers to give rise to shear-thickening; a second shear thinning region at high shear rates, where the shear stress is high enough to destroy the arranged WPU-SF composite particles; and finally a plateau region at higher shear rates, where the domains have all been oriented along the shear direction. For the viscosity profiles of WPU20-SF3 and WPU25-SF3 dispersions, the observed shear thinning is attributed to deformation and realignment of the gel domains. Figure 2f depicts the viscosity profiles of the WPU17-SF dispersions with different SF concentrations ranging from 1 to 5 wt %. All the WPU17-SF samples investigated exhibit the multi-distinct regions mentioned above. At low shear rates, a Newtonian plateau for viscosity is observed and the corresponding viscosity value increases with SF concentration. It indicates that the SF particles in the WPU dispersions provide interaction points and hence increase the density of entanglement structures in WPU-SF dispersions.

### 3.2. Dynamic Oscillation Behavior of WPU Dispersions

**Figure 3:**
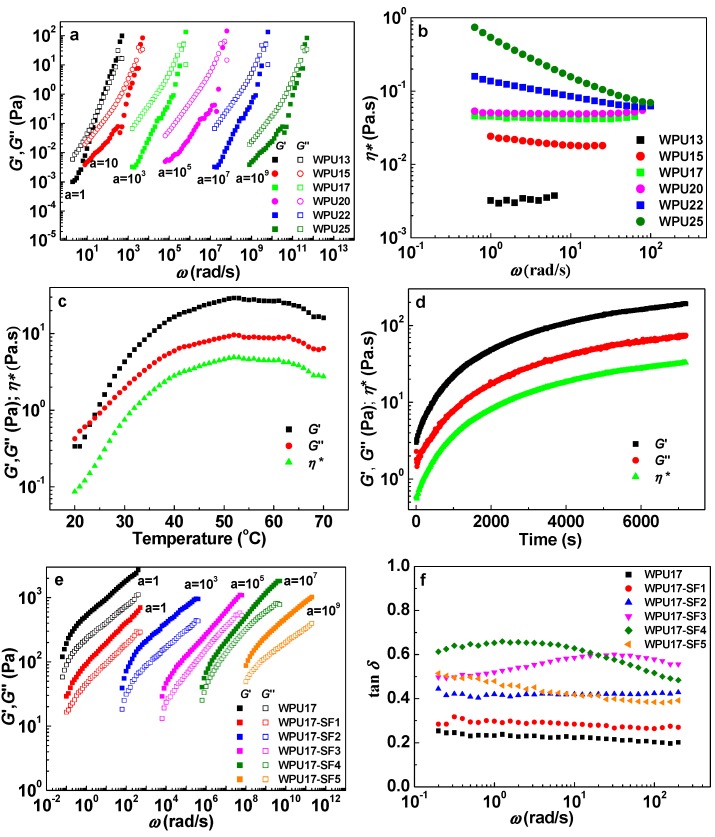
Dynamic rheological behavior of WPU and WPU-SF dispersions under oscillatory shear: (**a**,**b**) *G′* and *G*′′, and η* as a function of *ω* for WPU dispersions with different solid contents at *T* = 25 °C; (**c**,**d**) Temperature and time dependence of *G*′, *G*′′, and η* for the WPU17-SF5 dispersion; (**e**,**f**) Dynamic shear moduli, *G′*, and *G*′′, and loss tangent, tan *δ* as a function of ω for WPU17-SF dispersions with different silk fibroin contents at *T* = 37 °C. The x-axis of (**a**,**e**) is extended by a factors a = 1~10^9^ to obtain a valid comparison.

Figure 3a demonstrates the effect of WPU solid content on the dynamic shear moduli, *G*′ and *G*′′, at 25 °C. The *x*-axis was shifted to higher frequency range by a factor, a, ranging from 1 to 10^9^, as shown in the figure to obtain a valid comparison. The *G*′ and *G*′′ values of WPU dispersions were strongly frequency dependent for all the WPU dispersions with the solid contents in the range of WPU wt % from 13 to 25 in the frequency range investigated. For the WPU dispersion with the solid content of 13 wt %, *G*′ and *G*′′ curves intersected at the middle of the frequency range, exhibiting a rheological behavior same as a dilute solution. When the solid content of WPU dispersion is in the range from 15 to25 wt %, *G*′ value is lower than that of *G*′′ and parallels to each other at low frequency range, whereas the moduli approach each other at higher frequencies. It indicates that the dispersions behave as typical Brownian suspensions. The frequency-dependence of the dynamic complex viscosity, η∗, of WPU dispersion at different solid contents is shown in Figure 3b. The η∗ values increased abruptly with increasing solid content. For solid contents from 13 to 20 wt %, the viscosity was independent of frequency. The shear-thinning behavior was observed across the entire frequency range for WPU dispersions with the solid content of 22 and 25 wt %. The η∗ value of WPU dispersion with the solid content of 25 wt % was much higher compared to that of dispersions with lower solid contents at low frequencies, however, at high frequencies the η∗ value for the WPU dispersion with the solid content 17 to 25 wt % was close to each other. This can be due to the fact that the WPU dispersions at high solid contents, *i.e.*, at 22 and 25 wt % may form aggregates or gel-like network structures, which result in higher viscosity compared to that of lower solid content dispersions. The formation of aggregates or gel-like networks in dispersion system with solid contents more than 17 wt % may restrict the dispersion of SF particles into the WPU prepolymer matrix homogeneously in the WPU-SF dispersions. Therefore, the 17 wt % WPU dispersion was chosen in this study to further study the effect of SF on the properties of WPU-SF composites, which were prepared by adding SF powder into the reactor system of WPU17 prepolymer formulation.

### 3.3. Dynamic Oscillation Behavior of WPU17-SF Dispersions

Dynamic rheology is an effective method for studying the curing process and for monitoring gelation or crosslinking process of thermosetting polymers. To examine the effect of temperature on the properties of WPU-SF suspensions, temperature sweep was performed at 1 Hz. The changes of *G′*, *G*′′, and η∗ with the temperature for the WPU17-SF5 suspension are shown in Figure 3c. As temperature increased, a dramatic increase in *G′*, *G*′′, and η∗ is observed at the initial stage over the temperature range from 20 to 50 °C, and *G′* exceeds *G*′′ starting from 25 °C, which is attributed to the gelation process. The *G′*, *G*′′, and η∗ values reach a plateau region over the temperature range from 50 to 70 °C, which is a typical criterion for the formation of an elastic and fractal gel. For further monitoring the variation of *G′*, *G*′′, and η∗ as a function of curing time, time sweep for the WPU17-SF5 suspension was performed at 1 Hz at 37 °C over an extended period of time, and the result is depicted in Figure 3d. The elastic response is dominating (*G′* > *G*′′), and the moduli and complex viscosity increase rapidly from the beginning of the test, and slow down after approximately 30 min. It indicates that gel structures have formed in the WPU17-SF3 dispersion at 37 °C, and the gelation process can develop as time increasing. The storage modulus and loss modulus, *G′* and *G*′′, as functions of oscillatory frequency for WPU17-SF dispersions at 37 °C are shown in Figure 3e. The SF contents varied from 0 to 5.0 wt %, and both the *G′* (ω) and *G*′′ (ω) increased with the increase of ω. The first experimentally established scaling law *G′* (ω) = *G*′′ (ω) ~ω^1/2^ at the gel point, and later generalized it to be [33].

(2) *G′* (ω) = *G*′′ (ω) ~ω^n^

where the exponent *n* is the relaxation exponent at gel point, and is relative to microstructure parameters of gel system. The value of *n* varies from one system to another, and can range from 0 to 1 depending on the specific nature of the gelling system [34,35]. If the value of *n* is smaller than ½, the crossover of *G′* and *G*′′ happens before the gel point [33], while if the value is higher than ½ the crossover happens after the gel point. For *n* > ½, gel point occurs earlier than the crossover due to the imbalanced systems that are lean on cross-linker [35]. On the other hand, it has been reported that a high molecular weight prepolymer results in critical gels with low relaxation exponent values ranging from 0.2 to 0.4 [36]. The entanglement of molecules reduces the exponent value and makes the gel more elastic [37,38]. From the curves in Figure 3e, the *G′* (*ω*) ~*ω*^n′^, and *G*′′ (*ω*) ~*ω*^n′′^ relationships were fitted for the WPU17-SF dispersions, and the obtained values are listed in Table 2. It is apparent that the *n*′ and *n*′′ take similar values between 0 and 1, indicating the formation of a gel plateau, according to the method by Nijehuis and Winter [39]. The values of *n*′ and *n*′′ are in the range from 0.23 to 0.39, and are lower than ½, implying that an elastic and dense gel structure formed in the WPU17 and WPU17-SF dispersions. It has been reported that critical gels with high values of the relaxation exponent have low fractal dimensions and are said to be “open”, and a decrease in the value of the relaxation exponent suggests a gel with a more “tight” network structure [36].Furthermore, based on the variations of the excluded volume interactions, Muthukumar developed an analytical model that rationalizes values of the relaxation exponent *n* in the whole range of 0< *n* < 1 for a polydispersed material [40].

(3) $$n=\frac{d(d+2-2d_{f})}{2(d+2-d_{f})}$$

where *d_f_* is fractal dimension and *d* (*d* = 3) is spatial dimension. By substituting the *n* values in Equation (3), the fractal dimension can be calculated, and the results are listed in Table 2. The results show that the values of the fractal dimension are around 2.3 for the WPU17 and WPU17-SF dispersions. The slight decrease of *d*_f_ with the addition of SF may be attributed to the development of phase separation and the formation of more heterogeneous polymer network in the WPU17-SF dispersions compared to that in the neat WPU17 dispersion [41]. A simplified model based on classical rubber elasticity theory is used to estimate the average gel pore size, *i.e.*, the effective concentration elastic chain (N*) [42]. N* includes the effects of both covalent linkages and physical entanglements. The presence of the SF affects N* and can be analyzed based on the rubber elasticity theory, which states that N* (mol/m^3^) is related to the equilibrium shear modulus, *G*_e_ of the hydrogel according to [43].

(4) $$G_{e}=N*RT$$

where *R* and *T* are the Gas constant and the absolute temperature. We used the *G*′ values at 1 Hz during frequency sweep experiment to estimate the N*values of the WPU17-SF dispersions. Based on the *G*′ values and Equation (4), the N* values are estimated and listed in Table 2. As SF content increases, the effective concentration of elastic chain increases, indicating formation of relative denser crosslinking network.

The tanδ values at 25 °C for various WPU17-SF dispersions as a function of ω are shown in Figure 3f. As the solid content of SF increased, tanδ value also increased at all frequencies. The values of tanδ for WPU17, WPU17-SF1 and WPU17-SF2 were independent of frequency over the entire frequency range of 0.1 to 200 rad/s, whereas the WPU17-SF4 and WPU17-SF5 showed a constant value of tanδ at low frequency values, subsequently a continuous decrease with the increase of frequency. This indicates the formation of more elastic and stiffer gel structure in WPU-SF dispersions compared to that in the neat WPU17 dispersion [24].

### 3.4. Particle Size and Zeta Potential of the WPU17-SF Dispersions

The particle size of the WPU17 and WPU17-SF dispersions of various SF contents are listed in Table 2. The pure WPU17 dispersion exhibited a unimodal distribution pattern with an average particle diameter of 56.2 nm. As the SF content increased, the particle size also increased. Also, as the SF content ranged from 1 to 5 wt %, the distribution of the composite dispersions exhibits two peaks. This change possibly results from the conjugate reaction and hydrogen-bond interactions between WPU17 and SF, as several groups of the SF molecule, namely the *N*-terminal amine, the sulfhydryl group and the hydroxyl group are ready to react with the isocyanate group of the prepolyurethane [23,44,45]. Furthermore, prepolyurethane has several amide ester groups (–NHCOO–), which are capable of forming hydrogen bonds with the hydroxyl groups or carboxyl residues. Besides, the fibroin proteins, which are semi-crystalline biopolymers with a significant content of the organic compounds glycine, alanine, and serine, are essentially a group multi-block copolymers containing crystallizable and uncrystallizable blocks that occur naturally [21]. The semi-crystalline SF leads to a more regular structure of the hard segments of WPU17. It has been reported that polymer phase separation and the ordered packing of the hard segments is enhanced due to the interaction between the hard PU and Clay segments via their hydrogen bonds [18]. Therefore, the conjugated and hydrogen bond interactions and the increasing hard microdomains may be contributed to the formation of strong composite particles with relative large size in WPU17-SF dispersions.

The zeta potentials of the WPU17 and WPU17-SF dispersions as the SF content varies are also listed in Table 2. The WPU17 dispersion has a negative zeta potential of −32.0 mV. COO^−^ ions of neutralized DMPA are useful in stabilizing dispersed WPU17 particles. Therefore, the WPU17 particles thus demonstrated negative surface charges and zeta potential. The SF particles had a zeta potential value of −15.2 mV when dispersed in water [25], indicating that the SF particle surface has negative charges. The electrical repulsion of the surface charges keep the SF particles dispersed in WPU17 matrix as an individual sphere in a reasonable state of stability. As the SF content increased, the composite dispersions displayed more negative zeta potentials. This is attributed to the formation of larger colloidal particles through conjugate and hydrogen bond reactions between WPU17 prepolymer and SF during the synthesis process. This result is in harmony with the result from the analysis of particle size.

### 3.5. Structure and Properties of the WPU17-SF Films

**Figure 4:**
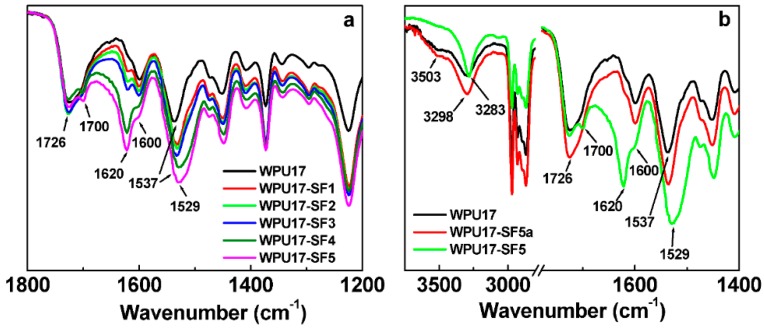
FTIR spectra of the WPU17, WPU17-SF and WPU17-SF5a films: (**a**) FTIR spectra of the WPU17 and WPU17-SF films in the wavenumber range of 1800–1200 cm^−1^; and (**b**) FTIR spectra of the WPU17, WPU17-SF5a, and WPU17-SF5 films in the wavenumber range of 3750–1400 cm^−1^.

The FTIR spectra of the WPU17 and WPU17-SF films are shown in Figure 4a. For the pure WPU17 film, the absorption peaks centered at 1726, 1600, and 1537 cm^−1^ are ascribed to the stretching vibration of the free C=O (C=O_free_) band, aromatic C=C band, and C–N stretching and N–H deformation vibrations, respectively, in the WPU17 [46,47]. Comparison of the spectra of the WPU17 and WPU17-SF, new absorption bands at 1620 and 1529 cm^−1^ appear, which correspond to the absorption of C=O and N–H bending deformation combined with C–N asymmetric stretching respectively [17,48]. It indicates that the tiny SF powder is covalently bonded to the WPU molecular chain during the synthesis of prepolymer through the reaction of hydroxyl groups on the surface of SF powder with isocyanate of WPU. The SF powder acts like a chain extender and cross-linking agent for WPU prepolymer. Furthermore, the appearance of the absorption band at 1700 cm^−1^ is due to the hydrogen bonded C=O (C=O_bonded_) stretching vibration, and the intensity ratio (C=O_bonded_/C=O_free_) increases with the increase of SF content. It indicates the existence of the strong hydrogen bonding between SF and WPU17.

For comparison and clarification, we prepared a WPU17-SF5a film with the same WPU to SF ratio as WPU17-SF5 but by mixing SF into WPU dispersion after the WPU dispersion was formed. The FTIR spectra of the WPU17, WPU17-SF5 and WPU17-SF5a films were shown in Figure 4b. For the WPU17 film, the weak and broad absorption peak centered at 3503 cm^−1^ is due to the free N−H (N−H_free_) stretching vibration, whereas the strong and sharp absorption peak at 3298 cm^−1^ arises from the hydrogen bonded N−H (N−H_bonded_) stretching vibration. Upon introduction of SF during the synthesis processing of WPU prepolymer, the N−H_bonded_ peak becomes sharper and shifts to a lower wavenumber from 3298 to 3283 cm^−1^, and no bands at about 3503 cm^−1^ are detected, indicating that all N−H_free_ groups are hydrogen-bonded in WPU17-SF films. Comparison with the pure WPU17, new absorption bands at 1700, 1620 and 1529 cm^−1^ are observed in the FTIR spectrum of the WPU17-SF5 film (explained as mentioned above), whereas almost no new absorption bands or peak shifts can be detected in the FTIR spectrum of WPU17-SF5a. It indicates that it is difficult to form interaction between SF and WPU by mixing 5 wt % SF into WPU dispersion after the WPU dispersion was formed. Thus, by *in situ* adding SF powder during the synthesis processing of WPU prepolymer, the SF powder can covalently bond to the WPU molecular chains and the hydrogen bonding formed between SF and WPU17.

**Figure 5:**
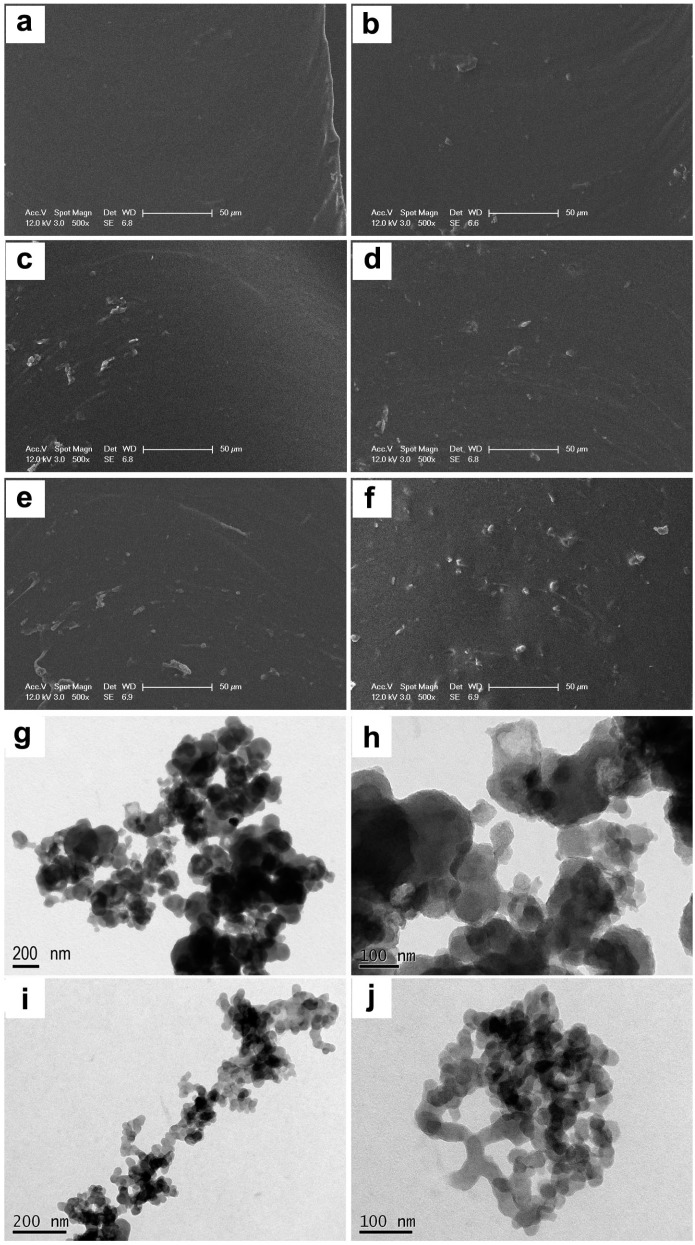
SEM and TEM images of the cross-sections of WPU and WPU-SF films: (**a**–**f**) SEM images of the WPU17, WPU17-SF1, WPU17-SF2, WPU17-SF3, WPU17-SF4, and WPU17-SF5 films in sequence; (**g**–**j**) TEM images of the WPU17-SF3 film.

The fractured surfaces of the WPU17-SF films were examined using an SEM. The SEM figures of the WPU17 matrix mixed with 0, 1, 2, 3, 4 and 5 wt % SF are shown in Figure 5a–f, respectively. As the SF content increased, a higher concentration of white dots appeared on the fractured surface of the composite films, indicating that the SF appears as white dots. SF aggregates were homogenously distributed in all blended films with WPU17 matrix, implying that SF particles disperse well in WPU17 matrix. This occurrence is attributable to the conjugate reaction as well as the hydrogen-bond interactions between SF and WPU. Furthermore, the homogenous distribution of SF in the WPU17 matrix is achieved also due to the negative charge of both the WPU17 and the SF particles.

Ultra-thin sections captured with the TEM of the WPU17-SF3 film are shown in Figure 5g-j. SF is dispersed in WPU17 matrix as groups with an average size of ca 480 ° 670 nm^2^, and the needle-shaped aggregates were also observed. Electrostatic interaction is considered to be responsible for the formation of the aggregates. Using the high magnification images obtained via the TEM of the WPU17-SF3 film, it is found that the microscale aggregates were consisted of nano-particles with sphere shape.

**Figure 6:**
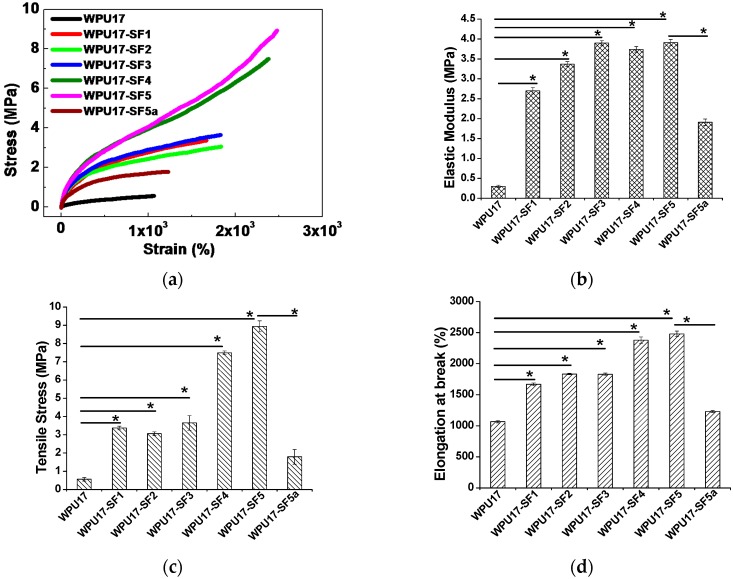
Uniaxial tensile mechanical properties of WPU17 and WPU17-SF films: (**a**) stress-strain curves; (**b**) elastic modulus; (**c**) tensile strength; and (**d**) elongation at break. WPU17-SF5a is the blended film with 5 wt % SF, which is prepared by blending SF into WPU17 dispersion, and is plotted for comparison. The bars represent mean ± standard deviation (*n* = 3; *p < 0.05).

**Figure 7:**
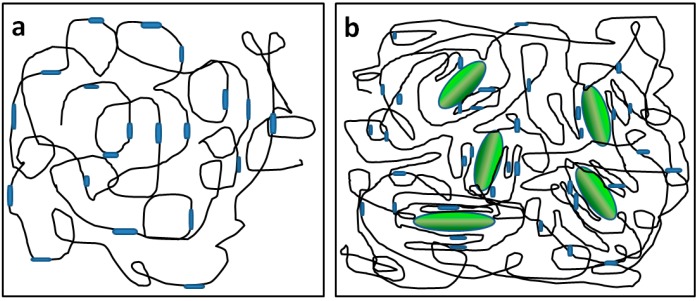
Schematic Illustration of Phase Separation in the: (**a**) WPU17 and (**b**) WPU17-SF films. The black and thin line, the blue and thick line, and the green ellipsoid represent soft segments of polyurethane, hard segments of polyurethane, and silk fibroin, respectively.

Information about the internal structure of the blended materials could be deduced from the mechanical properties of the material. Uniaxial testing was performed at room temperature to investigate the mechanical behavior of the WPU17 matrix with varying SF content. The stress-strain curves of the films obtained from the uniaxial tensile test are shown in Figure 6a. From the curves, we were able to determine the Young’s modulus (Figure 6b), tensile strength (Figure 6c) and the elongation at break (Figure 6d), respectively. The Young’s modulus was obtained from the initial slopes in the elastic region of the curves. From the curves, it is evident that WPU has a nonlinear elastic behavior, and has a Young’s modulus of 0.3 MPa, as well as a high elongation at break of about 1067%. From the tensile curves obtained, we can see that the presence of SF powder has a significant consequence on the tensile properties. The addition of a minor amount of SF significantly caused the tensile properties to improve. WPU17-SF films with 5 wt % SF content had significantly stronger Young’s modulus and tensile strength, about 13 times and 16 times stronger respectively than those of the pure WPU17 film. It should be noted that the incorporation of SF strengthens, stiffens, and toughens the WPU17 matrix in the range from 1 to 5 wt %, leading to an improvement in the mechanical properties studied (Young’s modulus, tensile strength, and elongation at break). The improvement in elasticity might be explained by the hypothesis that the SF increases the density of trapped entanglements between itself and the WPU17 molecules, acting like a cross-linking agent. The proposed mechanism for the significant reinforcement effect of SF powder in WPU17-SF films is shown schematically in Figure 7. Without SF, phase separation between the hard and soft segments in the WPU is minimal, and with the addition of SF powder into the reaction system during the synthesis of WPU prepolymer, the hydroxyl groups, amino groups, and carboxyl groups on the surfaces of the SF powders interact or react with the hard segments of the WPU prepolymer to form a much stronger network structures. Therefore, SF powder acts like a chain extender and cross-linking agent for WPU prepolymer. Furthermore, the crystal β-sheet structure of SF may induce the hard segments of WPU to arrange more regularly, which leads to further phase separation in WPU. Consequently, the hard micro-domains are more difficult to be stretched, leading to a much higher modulus, and the network structures enhance the elasticity.

In order to verify our hypothesis, we prepared a WPU17-SF5a film with the same WPU to SF ratio as in WPU17-SF5 but by mixing SF into WPU dispersion after the WPU dispersion was formed. The Young’s modulus, the tensile strength, and the elongation at break of the WPU17-SF5 film were much higher compared to those of the WPU17-SF5a film. This indicates that a strong interaction such as conjugated reaction or hydrogen bond between SF and prepolyurethane if SF is added during the WPU synthesis process. These strong interactions result in the formation of stronger network structure, which was contributed to improving the mechanical properties. These results are also in good agreement with those from the rheological properties, particle size analysis and zeta potential.

## 4. Conclusions

The WPU dispersion with the solid content of 13 wt % exhibits Newtonian behavior, and when the solid content increased from the 13 to 25 wt %, shear-thinning pseudo-plastic properties were observed. The gel network formed when the solid content was higher than 17 wt %. Therefore, 17 wt % WPU was chosen as a matrix to study the effect of silk fibroin powder on the rheological behavior, structure, and mechanical properties of WPU. The SF powders were well dispersed into the WPU17 matrix, and the negative surface charge played important role in maintaining the stability of the WPU17-SF dispersions with larger particle size. The hydrogen bond interactions between the urethane groups of WPU and hydroxyl and carboxyl groups on the surface of SF powder, and conjugate interaction between SF and WPU prepolymer contributed to formation of stronger cross-linked network. Furthermore, the β-sheet structure of silk fibroin encouraged more ordered arrangement of the hard segments of WPU. The formation of stronger cross-linking network and more ordered pack of hard segments led to the improvement of mechanical properties. Thus significant improvements in the properties and behavior of WPU dispersions and films were achieved by introducing SF powder into the WPU formulations and thereby forming novel WPU-SF composites. These WPU-SF composites can be very useful in the field of tissue engineering as the matrix material for 3D cell culture, to a sturdy drug carrier in specific regenerative medicine applications due to its aqueous processability, biocompatibility, and biodegradability.

## Figures and Tables

**Table 1:** Formulation of the waterborne polyurethane (WPU17) and WPU17-silk fibroin (WPU17-SF) dispersions.

Samples	Weight of Raw Materials (g)						SF Content (wt %)
	SF	PPG	DMPA	TDI	TEA	H_2_O	
WPU17	0	23.60	1.58	7.20	1.20	150	0
WPU17-SF1	0.34	23.60	1.58	7.20	1.20	150	1
WPU17-SF2	0.68	23.60	1.58	7.20	1.20	150	2
WPU17-SF3	1.01	23.60	1.58	7.20	1.20	150	3
WPU17-SF4	1.35	23.60	1.58	7.20	1.20	150	4
WPU17-SF5	1.68	23.60	1.58	7.20	1.20	150	5

**Table 2:** The Relaxation Exponent *n*′ and *n*′′ at the Gel Point, the effective concentration elastic chain (N*), Particle Size, and Zeta Potential of the WPU17 and WPU17-SF Dispersions.

Samples	*n*′	*d_f_* ^a^	*n*′′	*d_f_* ^b^	N* (mol/m^3^)	*D_h_* (nm)	ξ (mV)
WPU17	0.27	2.35	0.26	2.36	0.205	56.2	−32.0 ± 1.8
WPU17-SF1	0.32	2.32	0.31	2.33	0.036	63.6	−36.6 ± 4.4
WPU17-SF2	0.27	2.35	0.27	2.35	0.081	93.8	−38.4 ± 4.8
WPU17-SF3	0.35	2.30	0.37	2.29	0.048	111.5	−51.5 ± 6.4
WPU17-SF4	0.39	2.27	0.35	2.30	0.081	133.9	−53.7 ± 9.0
WPU17-SF5	0.27	2.35	0.23	2.38	0.095	112.8	−40.5 ± 5.7

*d_f_*^a^ values were calculated from *n*′; *d_f_*^b^ values were calculated from *n*′′.
